# The Polarizing Trend of Regional CO_2_ Emissions in China and Its Implications

**DOI:** 10.1021/acs.est.2c08052

**Published:** 2023-02-28

**Authors:** Kehan He, Zhifu Mi, Jin Zhang, Jinkai Li, D’Maris Coffman

**Affiliations:** † The Bartlett School of Sustainable Construction, University College London, London, WC1E 7HB, U.K.; ‡ Center for Energy, Environment & Economy Research, Zhengzhou University, Zhengzhou 450001, China; § School of Public Policy and Management, Tsinghua University, Beijing 100084, China; ∥ Center for Energy Economics and Sustainability, Peking University, Beijing 100871, China

**Keywords:** Input−Output Model, CO_2_ Emission Inequality, Regional Economies, Emission Outsource, Changing Emission Trends

## Abstract

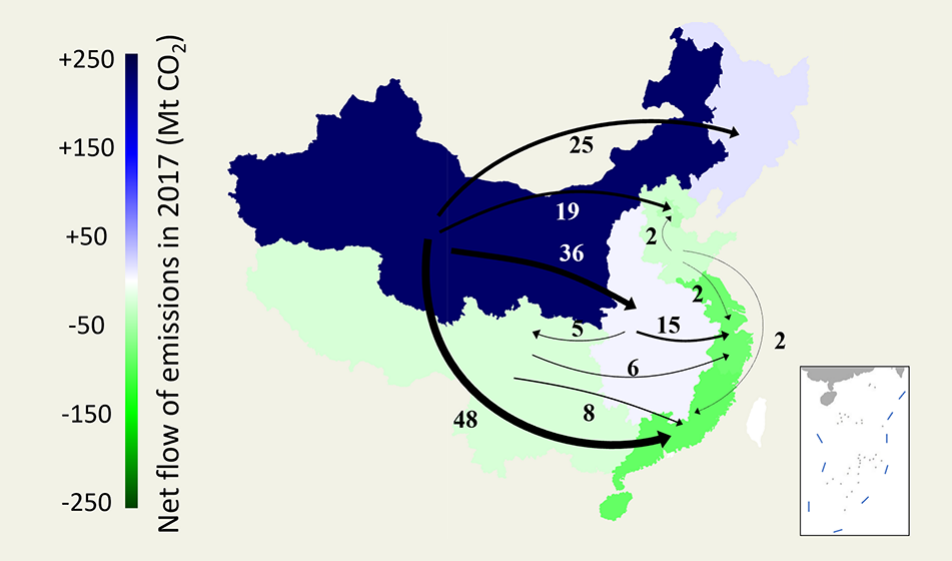

CO_2_ emissions are unevenly distributed both globally and regionally within nation-states. Given China’s entrance into the new stage of economic development, an updated study on the largest CO_2_ emitter’s domestic emission distribution is needed for effective and coordinated global CO_2_ mitigation planning. We discovered that domestic CO_2_ emissions in China are increasingly polarized for the 2007–2017 period. Specifically, the domestically exported CO_2_ emissions from the less developed and more polluting northwest region to the rest of China has drastically increased from 165 Mt in 2007 to 230 Mt in 2017. We attribute the polarizing trend to the simultaneous industrial upgrading of all regions and the persistent disparity in the development and emission decoupling of China’s regions. We also noted that CO_2_ emissions exported from China to the rest of the world has decreased by 41% from 2007 to 2017, with other developing countries filling up the vacancy. As this trend is set to intensify, we intend to send an alarm message to policy makers to devise and initiate actions and avoid the continuation of pollution migration.

## Abstract 2

Our study reveals the widening CO_2_ emission inequality of regions in China and the emerging trend of CO_2_ emissions to other emerging economies.

## Introduction

Anthropogenic CO_2_ emissions are driven by economic activities. As a result of differentiated levels of economic development, the CO_2_ emissions embodied in economic consumption, or consumption-based accounting (CBA) emissions, are distributed unequally across the globe. From the global perspective of regional economic development, CO_2_ emissions have increasingly shifted from developed regions to developing regions, whose population generally earns lower incomes.^1^ Recent studies confirm that CO_2_ emissions have been relocating to developing regions with increasing speed.^2−4^ Since the start of the new millennium, the CO_2_ emissions produced by developing regions have drastically increased compared to those produced by developed regions.^5^ On the other hand, some developed regions of the EU and North America have already achieved a decoupling of CO_2_ emissions and economic growth.^3^ However, this much-lauded decoupling has often been achieved at the expense of exploiting the emissions embodied in imports from developing regions.^6^ Research shows that the emissions embodied in trade from developing regions to developed regions have increased drastically from 0.9 Gt CO_2_ in 1996 to its peak of 2.1 Gt CO_2_ in 2006, although they then quickly decreased to 1.5 Gt in 2016.^7,8^ In addition, it is likely that poverty alleviation efforts will mean that those newly lifted out of poverty and near-poor individuals will increase their demands on energy consumption.^9^ Thus, poverty alleviation in developing regions can inadvertently contribute to intensified CO_2_ emissions.^10,11^

Being the single largest CO_2_ emitter, China has been studied by many for its consumption-based CO_2_ emissions. As a net exporter of CO_2_ emissions,^12,13^ China’s success at economic upgrading decreased its emissions embodied in exports from 2008 to 2015.^14^ Due to its economic and geographical size, China’s provinces remain varied in their levels of development. Thus, domestic trade-embodied emissions and their associated energy consumption are also considered a key research topic.^15^ Recent research has identified that China’s western regions are net domestic exporters of embodied CO_2_ emissions to coastal eastern regions due to the differences in China’s domestic economic structure and development.^16−19^ Some lately published research accounts for the CO_2_ CBA of China’s provinces in 2017.^20,21^ However, none of the referenced studies emphasize the intensifying domestic inequalities in consumption-based CO_2_ emissions among China’s regions, as well as the possibility of their further development. Since China entered the so-called economic “new normal” in 2012,^22^ the economic and CO_2_ emission structures may have undergone alterations. Economic transformation and development in China may also imply that other countries may take up the polluting roles in the coming years. Research is thus needed to characterize and understand the rationale and scale of these changes, thus formulate further policy recommendations in accordance with the widening inequalities and overseas outsourcing trend of CO_2_ emissions in China.

In this study, we used the latest available Multi-Regional Input–Output (MRIO) table for 2017 to reveal the latest trend in the inequal geographical distribution of consumption-based CO_2_ emissions in China by extending the investigation beyond 2012. The results of this research show that the inequality in the geographical distribution of CO_2_ emissions has intensified. Compared to previous years, CO_2_ emissions are polarized toward the less developed and emission-inefficient northwest region. With referenced and original evidence and supported by economic theories, we further argue that the reason for such polarization is the result of the lagged correlation between economic development and carbon emission efficiency, creating a so-called “carbon leakage” within China. In other words, some developed regions in China have achieved emission decoupling with economic growth like many other developed countries, while the less developed parts of China are still growing their economies at the expense of increased emissions. As China grows in economic strength while setting ambitious CO_2_ emission mitigation objectives, the outsourcing of China’s emissions to less developed and emerging economies should be a growing concern for global collaboration on carbon mitigation, as this dynamic duplicate concerns about the decoupling and cross-border outsourcing of emission flows in other parts of the world.

## Methods and Materials

### Consumption-Based Emissions

CBA allocates emission responsibilities to consumers. Unlike production-based accounting (PBA), where emissions are registered within territorial boundaries, CBA offers a lens to examine the emissions embodied in the upstream of products’ final destinations.^23^ Thus, by comparing the CBA for CO_2_ emissions, consuming provinces’ polluting responsibilities can be quantitatively analyzed to reveal the evolving trends in the inequalities of CO_2_ emissions among Chinese provinces. It should also be noted that the CBA for CO_2_ emissions mentioned in this study are all domestic CBA emissions, meaning that CBA emissions from overseas are simply removed as they are out of the scope in this study.

In CBA, calculation is based on Environmentally Extended Input–Output Analysis (EE-IOA),^24^ a widely adopted extension of the classic Leontief Input–Output Model.^25^ Originally, the Leontief Input–Output Model could be given by eq 1:
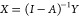
1

In eq 1, *X* is a vertical vector that denotes the total output by sectors and regions, and *Y* is a vertical vector that denotes the total final consumption by sectors and regions. *I* is an identity matrix that has ones on its diagonal and zeros as all the other elements. *A* is the production coefficient matrix, which shows the technical input needed per unit output.

The environmental extension of Input–Output Model and, thus, CBA calculation require a CO_2_ emission intensity horizontal vector *E* to be added so that we have eq 2 below:
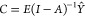
2

In eq 2, *C* is the CBA for CO_2_ emissions by regions and sectors given as a horizontal vector. *Ŷ* is the diagonalized form of *Y*.

Conventionally, there are two types of MRIO tables used by researchers. The noncompetitive MRIO table differentiates domestic and foreign intermediate productions as the production technical coefficients are separately given. On the other hand, competitive MRIO tables do not make such differentiation, but instead presents import from and export to other countries as separate columns. In this study, we adopt the noncompetitive MRIO table for calculations in accordance with the past studies. As the focus of this research is the domestic CO_2_ emission of China, we do not consider the import from and export to countries overseas by leaving out the export and import column of the competitive MRIO table of China.

### Emission Gini Coefficient

Economists often use the Gini coefficient to quantitatively compare income inequalities.^26^ Recently, some researchers have altered the methodology for calculating Gini coefficients to investigate the CO_2_ emission inequalities across different income groups.^27,28^ Here, in this research, we further changed the variables in the Gini coefficient calculations to directly show the difference in CO_2_ emissions among Chinese provinces instead of population groups. Originally, Gini coefficient is derived from the Lorenz Curve. The larger the Gini coefficient is, the more unequally the income is distributed among the population. In a Lorenz Curve, the horizontal axis is the fraction of population, while the vertical axis is the cumulative share of income. A line of equality indicates perfectly equal distribution of income among all the population. Denoting the area between the Lorenz Curve and the line of equality as *A* and the area between the Lorenz Curve and the axes as *B*, the Gini coefficient is simply given by *A*/(*A* + *B*). The emission Gini coefficient in this study changes the horizontal axis to the proportion of final consumption in China’s provinces and the vertical axis to the cumulative consumption-based CO_2_ emissions, as shown in Figure 2. Hence, the alternative version of the Gini coefficient can be calculated using eq 3 below:
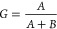
3

In eq 3, *A* is the area between the emission Lorenz Curve and the line of equality. *B* is the area between the emission Lorenz Curve and the axes. By changing the concept of the Gini coefficient into the format presented in eq 3, we intend to reveal the inequality in emissions embodied in consumption activities across Chinese provinces.

### Data

The MRIO table was compiled using 2017 Chinese provincial Input–Output tables published by the National Bureau of Statistics. Excluding regions and territories with no data available, we compiled 31 regions and 42 economic sectors in the 2017 Chinese MRIO table. It should be noted that the 2007 and 2012 Chinese MRIO tables have 30 regions and 30 economic sectors. The missing region in 2007 and 2012 is Tibet due to missing data. Since Tibet only composes a very small portion of consumption-based CO_2_ emission (0.06%) and final consumptions (0.25%) in China, we have included Tibet in the result of 2017 despite the inconsistency with past results to maximize the information presented in this study. Besides, although the number of economic sectors for the year 2007 and 2012 is 30, inconsistent with the 42 sector specification for the year 2017, we have aggregated them into single region for final result presentation. Thus, the issue of an inconsistent number of economic sectors is also resolved in this study.

In the construction of the 2017 Chinese MRIO table, we refer to the method utilized by Mi et al.,^22^ where the gravity model is applied to simulate interprovincial and intersectoral trade. The gravity model considers the trade between two locations to be directly proportional to the economic sizes of and inversely proportional to the distance between the two locations. Concretely, it can be expressed by eq 4:
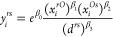
4

In eq 4, *y*_*i*_^*rs*^ represents the economic quantity of item *i* traded from location *r* to location *s*. *x*_*i*_^*rO*^ is the quantity of item *i* exported by location *r.x*_*i*_^*Os*^ is the quantity of item *i* imported by location *s.d*^*rs*^ is the distance between locations *r* and *s*. In this study, we used the distances of provincial capitals for *d*^*rs*^. β_1_, β_2_, and β_3_ are the model coefficients to be obtained through regression. *e*^β_0_^ is the error term. To reconcile for linear regression, eq 4 is manipulated into eq 5, as shown below:
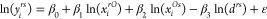
5Having the regressed coefficients, it is thus possible to model the economic flow between any two provincial sectors.

In addition to the standard gravity model, we also introduced impact coefficients to model the cooperative and competitive relationships among provincial sectors, which is given as *c*_*i*_^*gh*^ below in eq 5:
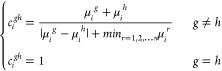
6

In eq 6, *c*_*i*_^*gh*^ is the impact coefficient for item *i* between locations *g* and *h* for *n* locations, which is the number of provinces in this case. It measures the strength of interaction of item *i*. μ_*i*_^*g*^ and μ_*i*_^*h*^ are the location quotients of item *i* in locations *g* and *h*.

Then, the trade flow obtained from the gravity model is further modified into eq 7 to reflect cooperative and competitive relationships using impact exponents *δ̅* – *δ*_*i*_:
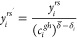
7

*δ*_*i*_ is the proportion of the total output of item *i* that it uses as its own intermediate inputs, while δ̅ is its average value. Hence, the denominator of eq 7 will adjust the trade flow modeled from the standard gravity model to reflect cooperation and competition. The final MRIO table is obtained with the RAS algorithm to ensure its consistency in column and row sums^29^.

## Results

**Figure 1:**
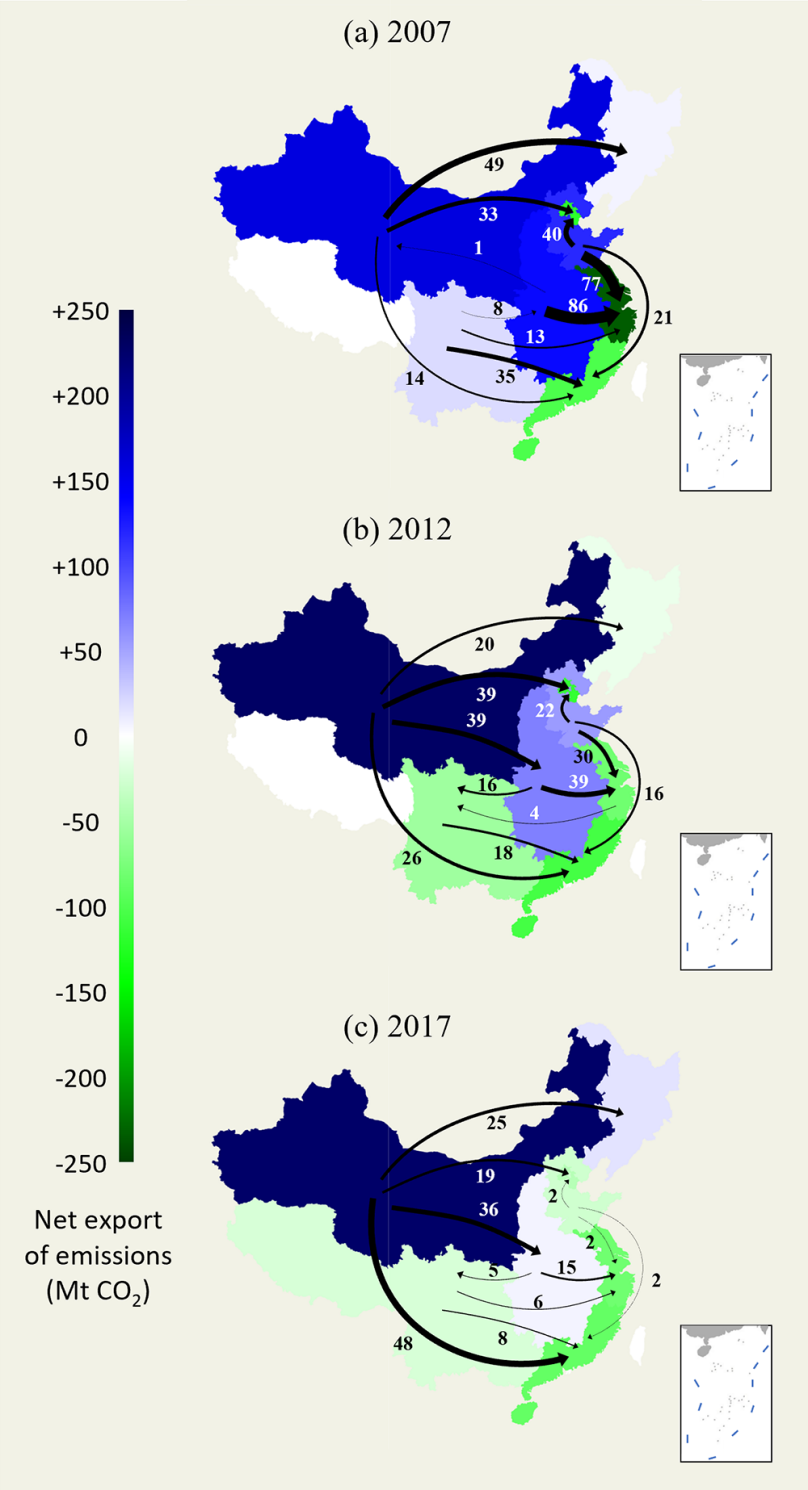
Net flow of CO_2_ emissions embodied in domestic trade among regions of China in (a) 2007, (b) 2012, and (c) 2017. Provinces colored in white indicate an absence of data. The 8 regions are divided in accordance with the widely practiced China administrative region specification (see Table S1 in Supporting Information). Note that not all transregional flows are presented in this figure due to artistic constraints. Please refer to the Table S2 in Supporting Information for the comprehensive data set used to produce this figure.

Our latest results for 2017 suggest that CO_2_ emissions in China continue to be shifted toward the less developed northwest region, creating a widening inequality in consumption-based CO_2_ emissions. In Figure 1, we organize China’s provinces into 8 identical regions to show the flow of CO_2_ emissions. From 2007 to 2017, the number of net CO_2_ emission domestic exporting regions decreased from 5 to 3. Specifically, the southwestern region first changed from a net exporter to a net importer in 2012 and remained a net importer in 2017, although its amount of CO_2_ emissions net imported decreased from 54 Mt to 22 Mt. Among all trade partners of the southwestern region, the northern and central coastal regions changed from net exporters in 2012 to net importers in 2017, suggesting strengthened industrial linkages among the newly developed regions of China. The same reverse for the northern region occurred later in 2017, with its CO_2_ emissions embodied in net exports decreasing from 60 Mt to −26 Mt. The largest decrease in CO_2_ emissions embodied in exports from the northern region occurred in the central coastal region from 2012 to 2017 (30 Mt to 2 Mt). Although a reverse has yet to be observed in the central region, a continuous and drastic decrease in net CO_2_ emission exports can be easily seen from 2007 to 2017. From 2012 to 2017, the net exports of embodied CO_2_ emissions greatly decreased from 71 Mt to 10 Mt. The central coastal region had the largest decrease (24 Mt) in net imports of CO_2_ emissions from the central region from 2012 to 2017. CO_2_ emission exports were also observed to be polarizing toward the northwest region. Specifically, after the sharp increase in 2012, the northwest region’s net exports of embodied CO_2_ emissions remained constant at 230 Mt, significantly outpacing the next net exporters, the northeast region (18 Mt) and the central region (10 Mt). This finding means that the northwest region is the only significant CO_2_ exporter among all eight regions of China, which is a very different situation than in 2007 and 2012, where the north and central regions also played significant roles in producing CO_2_ emissions for other regions of China. The northeast region showed an exceptionally volatile trend, meaning it experienced two reversals in net CO_2_ emission exports in 2012 and 2017. Another interesting observation is that although the Beijing–Tianjin, central coastal and southern coastal regions remain net CO_2_ emission importers, the quantities of embodied CO_2_ imported decreased by 78 Mt, 154 Mt, and 11 Mt, respectively, in 2017 compared to 2007, suggesting that their reliance on the domestic supply chain for pollution outsourcing decreased.

**Figure 2:**
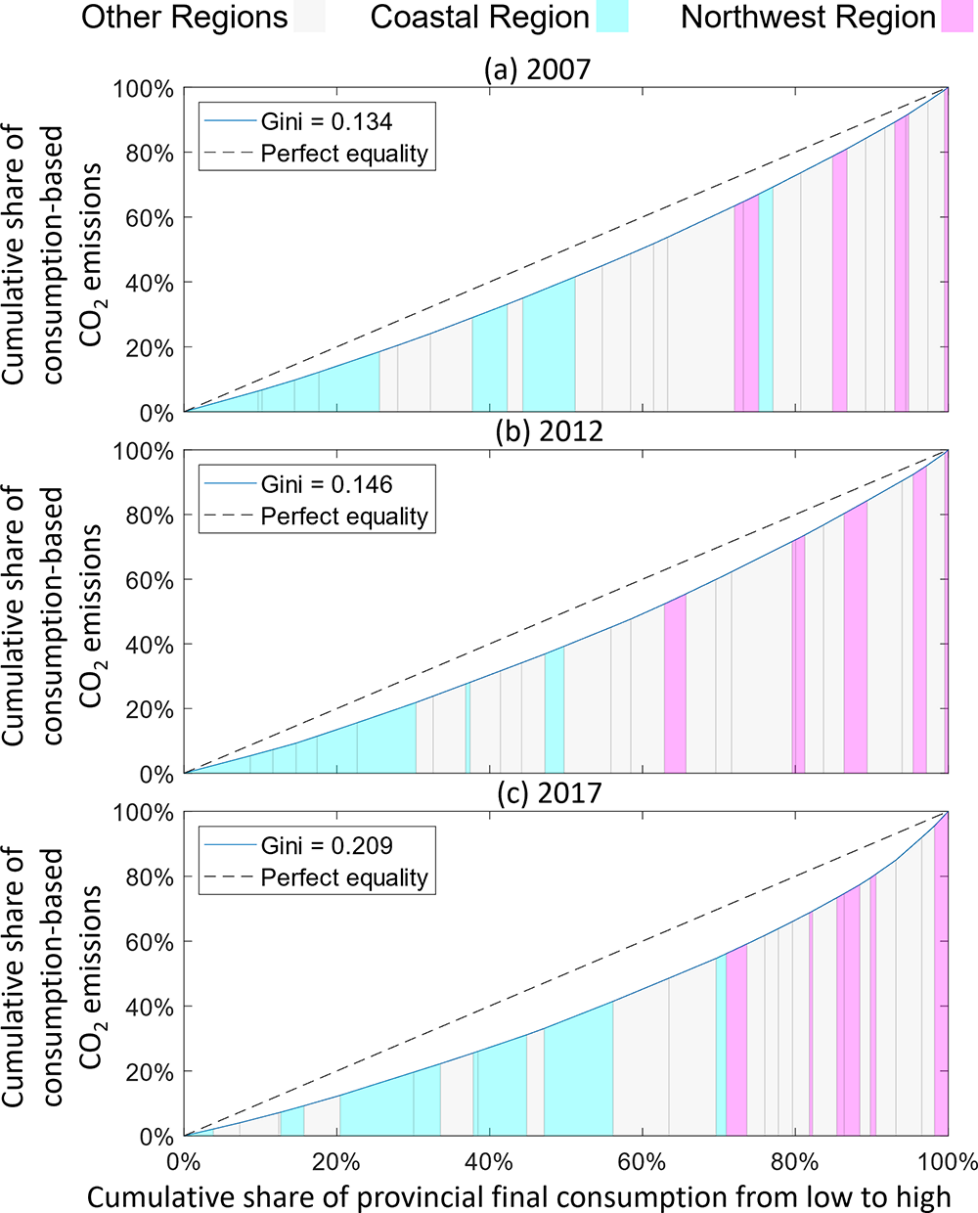
Lorenz curves of Chinese provincial consumption-based CO_2_ emissions in 2007, 2012, and 2017. The Lorenz curves have been altered so that the horizontal axis is the cumulative share of provincial final consumption and the vertical axis is the cumulative share of consumption-based CO_2_ emissions, i.e., each bar under the Lorenz curve has its width (horizontal axis) representing the amount of the province’s final consumption, while the height (vertical axis) representing the amount of emissions produced cumulatively added with the emissions produced by provinces positioned on its left. Provinces are positioned from left to right in ascending order of emissions produced.

Since the CO_2_ emissions of China have been polarized toward the northwest region, this phenomenon suggests that the inequality in the geographical distribution of emissions is intensifying. We thus introduced a modified emission Gini coefficient to quantitatively compare the emission inequalities among China’s provinces for the years 2007, 2012, and 2017. Instead of showing the distribution of income among populations, the modified emission Gini coefficient shows the distribution of consumption-based CO_2_ emissions among final consumption across provinces. Figure 2 shows the Lorenz curves of consumption-based CO_2_ emissions against the proportion of final consumption in China’s provinces from 2007 to 2017. It is clearly shown that the emission Gini coefficient among China’s provinces has drastically increased from 0.134 in 2007 to 0.209 in 2017. The reason for this change can be attributed to the different strength in CO_2_ emission decoupling between the developed and developing regions in China, which is discussed in more detail later in the Discussion section. In addition, we see that although an increase in emission inequality occurred from 2007 to 2012 (+0.012), it was not tantamount to the intensification of emission inequality from 2012 to 2017 (+0.063). It shows that the emission inequality is much widened between 2012 to 2017, implying that the more developed regions have been gradually reaching emission decoupling. It coincides with the emphasis of green development by the Central Government of China in more recent years, suggesting that the developed regions are more capable in responding to the policy shift as they possess more resources to do so. The Lorenz curve for 2017 is distorted toward the right, suggesting that CO_2_ emissions are more concentrated toward the northwest region.

**Figure 3:**
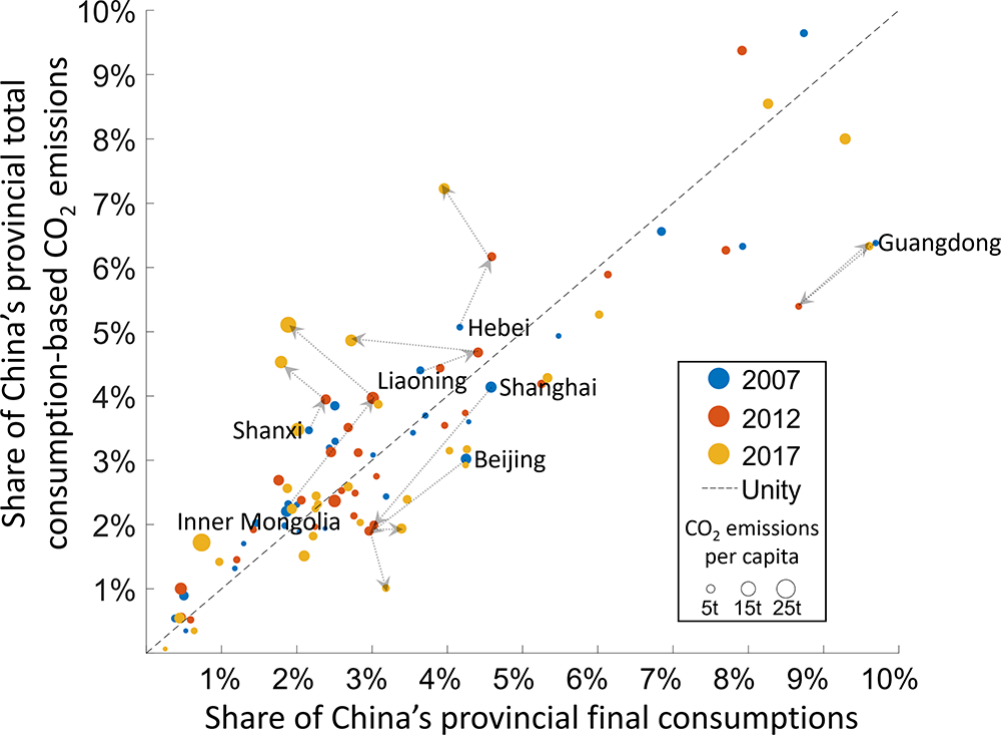
Shares of consumption-based CO_2_ emissions versus shares of the final consumption of Chinese provinces in 2007, 2012, and 2017. The sizes of dots are the consumption-based CO_2_ emissions per capita. Please see Table S4 in Supporting Information for data result.

The unequal distribution of emissions in China can also be shown by the disparities between final consumption and consumption-based CO_2_ emissions among provinces. While some developed provinces enjoy high levels of consumption, the CO_2_ emissions associated with them are disproportionately lower. Figure 3 is produced to make a convenient comparison of the two quantities. In Figure 3, the provinces on the left of the unity line induced more CO_2_ emissions than they consumed, and vice versa for the provinces on the right of the unity line. Observation shows that Inner Mongolia, one of the northwest provinces, is a typical province with disproportionately higher CO_2_ emissions than its consumption. In Inner Mongolia, the differences between the percentages of consumption-based CO_2_ emissions and final consumption increased by 2.8 percentage points from 2007 to 2017, the largest increase among all Chinese provinces, followed by Hebei (2.4), Shanxi (1.4), and Liaoning (1.4). The developed provinces of Guangdong, Beijing, and Shanghai, on the other hand, have a higher proportion of final consumption than consumption-based CO_2_ emissions. For Guangdong, the differences between the percentage of final consumption and consumption-based CO_2_ emissions remained at 3.3 percentage points from 2007 to 2017, but gradual increases in differences can be identified in other developed provinces, such as Beijing (0.9) and Shanghai (1.0). Moreover, increases in the disparities between the percentage of final consumption and consumption-based CO_2_ emissions can be seen in many more provinces. In Figure 3, scattered dots for 2007 are located closer to the unity line compared those for 2017, meaning that the disparities were more severe in 2017 than they were ten years prior. Another indicator suggesting a widening inequality is the number of provinces with a higher proportion of consumption-based CO_2_ emissions than final consumption. Provinces with a higher proportion of consumption-based CO_2_ emissions than final consumption generally have higher emissions per capita. The extent of the differences in emissions per capita also intensified in 2017 compared to 2007. This again indicates a polarizing trend of consumption-based emissions toward the less developed provinces of China.

**Figure 4:**
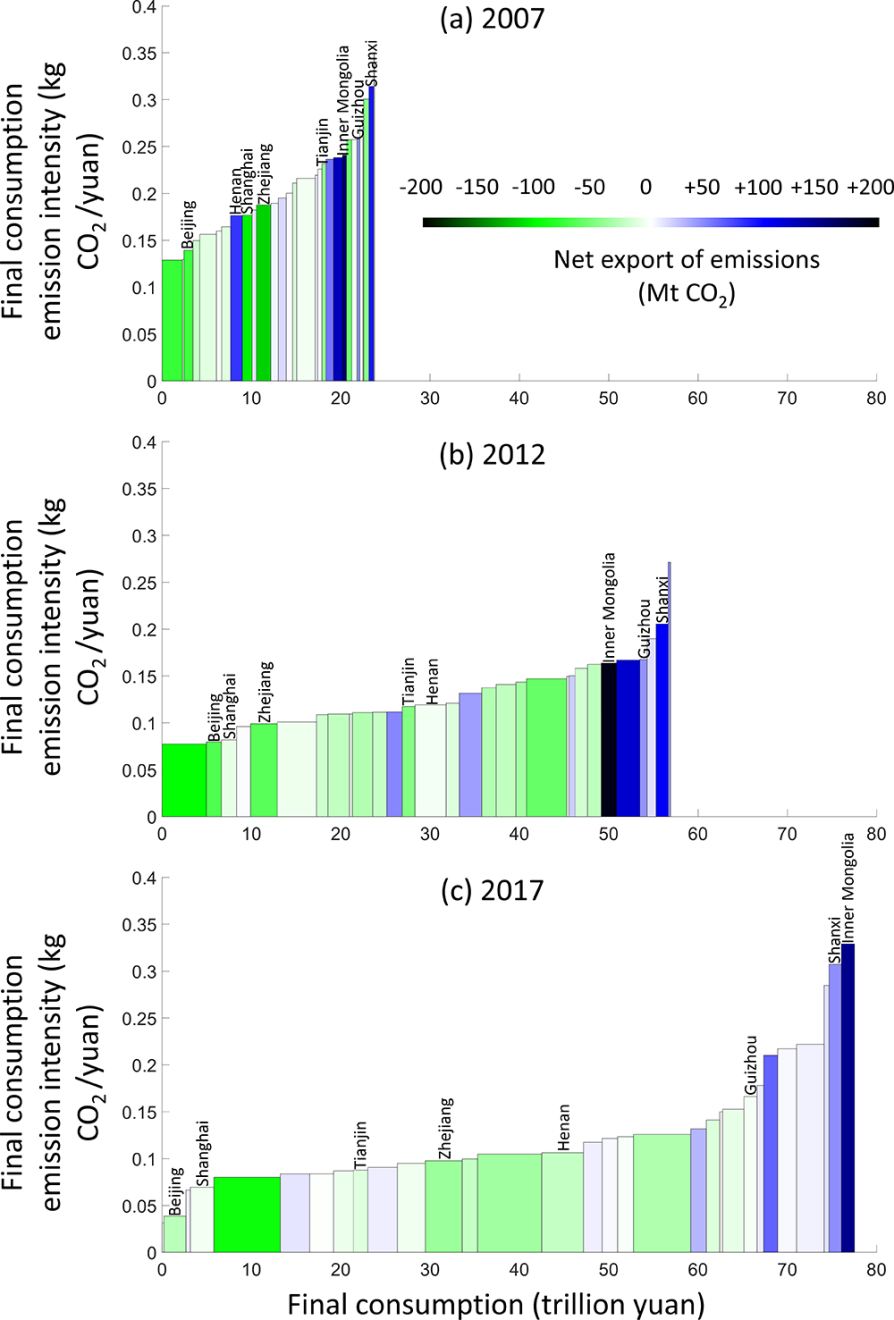
Final consumption, consumption-based CO_2_ emission intensities, and net exports of embodied CO_2_ emissions of China’s provinces in (a) 2007, (b) 2012, and (c) 2017. Each rectangular bar represents the size of the consumption-based emissions of the labeled province. The heights and widths of the bars show the final consumption emission intensities and final consumption of the labeled provinces, respectively. The face color of the bars indicates net trades of embodied CO_2_ emissions. Darker green means larger net imports of embodied CO_2_ emissions. Darker blue means larger net exports of embodied CO_2_ emissions. Please see Table S3 in Supporting Information for data result.

The discrepancy in consumption and consumption-based CO_2_ emissions can be analyzed based on the differences in emission intensity and emissions embodied in domestic trade, as shown in Figure 4. In general, provinces with higher emission intensities exported more embodied CO_2_ emissions to other provinces but caused less consumption-based CO_2_ emissions in 2017. The opposite applies for provinces with lower emission intensities. In 2017, all 5 provinces with the highest CO_2_ emission intensities were net exporters of embodied CO_2_ emissions. However, having high emission intensities before 2017 was not equivalent to being a net exporter of embodied CO_2_ emissions. Among the 5 provinces with the highest CO_2_ emission intensities in 2007, 3 were net importers. Inner Mongolia is the only province with an increased CO_2_ emission intensity, while it also remains the largest net exporter of embodied CO_2_ emissions from 2007 to 2017. For developed provinces such as Beijing, Shanghai, Tianjin, and Zhejiang, net imports of embodied CO_2_ emissions continuously decreased, with reductions amounting to 48 Mt, 100 Mt, 30 Mt, and 78 Mt, respectively, from 2007 to 2017. In contrast, the net exports of embodied CO_2_ from developing provinces, such as Hebei, Henan, Shanxi, and Guizhou also drastically decreased from 2007 to 2017, amounting to 123 Mt, 103 Mt, 48 Mt, and 46 Mt, respectively. Nevertheless, as the largest net importer and exporter of embodied CO_2_ emissions in 2017, Guangdong and Inner Mongolia (96 Mt imported and 146 Mt exported embodied CO_2,_ respectively) did not undergo much change in their trade-embodied CO_2_ emissions from 2007 to 2017 (differences of 17 Mt and 13 Mt, respectively). Thus, although the standard deviations of trade-embodied CO_2_ emissions decreased by 38% from 2007 to 2017, the outlying data points of Inner Mongolia and Guangdong again indicate a polarizing trend of the distribution of consumption-based CO_2_ emissions among provinces in China. This polarizing trend can also be visually deduced from the increased concavities of the plots in Figure 4. The tops of the rectangles are straighter in 2007 than in 2017, suggesting that the differences between emission intensities of various provinces increases faster in 2017 than in 2007. It is another indicator to show the polarizing trend of inequality in CO_2_ emissions.

## Uncertainty

Same to any research, this study is prone to limitations and weakness. Although economic factor is fundamentally determining to consumption level and hence consumption-based emission, there are other factors that play important roles in determining the consumption based CO_2_ emissions across geographical locations. For instance, provinces in the north requires more heating in colder seasons, which may contribute to higher consumption-based CO_2_ emissions as economic grows and residents’ income increases. As the degree of income elasticity varies for different factors, the extent on the noneconomic factors’ impact on consumption-based emissions and their inequalities may be investigated in further studies.

In addition, due to differences in specifications and data source used for MRIO table compilation, uncertainties may also arise between MRIO tables used and hence the calculated consumption-based emissions. We have performed the same calculation for consumption-based CO_2_ emissions using another recently published 2017 China MRIO table compiled by CEADs.^35^ Calculation with the alternative 2017 China MRIO table verifies the polarizing trend discovered in this study. For the consumption-based CO_2_ emissions of the 31 provinces, the average difference is 22%. If the two outlying results are removed, the average in differences is further reduced to 14%. It suggests the results in this study are generally accurate and reliable, but further investigations may be needed to discuss the specific provinces that have larger discrepancies in the CO_2_ emissions calculated.

## Discussion

In this study, we depicted the changing distribution of consumption-based CO_2_ emissions among Chinese provinces from 2007 to 2017. The general trend of consumption-based CO_2_ emissions flow is from inner lands to coastal regions, same as the most recent study of Dong et al.^21^ has found. Being more unique and focused, our result revealed that the unequal geographical distribution of CO_2_ emissions intensified. Emission responsibilities shifted toward a few provinces, which were mostly net exporters of embodied emissions to other provinces. In other words, less developed provinces are becoming the so-called “pollution haven” for the more developed provinces. In past studies, the hypothesis of a “pollution haven” has been proven with evidence at the international scale.^36,37^ Our study quantitatively tells the interesting story that the domestic transfer of embodied pollution in China not only exists but has also intensified in line with global trends. On the other hand, the consumption-based CO_2_ among provinces in China also shows a polarizing trend. More and more consumption-based CO_2_ emissions are now induced by the less developed regions in China, shown by the intensifying emission Gini coefficient. In the early 2000s, the coastal regions of China economically benefited from globalization and China’s opening up, constituting the first batch of developed Chinese regions. One possible explanation for the observation is the Flying Geese Paradigm proposed by Akamatsu.^38^ After acting as the outsourcing hub for other developed economies, the lower-end and more polluting industries of the coastal regions were phased out and moved to the less developed inland regions once industry upgrading was completed.^39,40^ This can also be explained by the “carbon leakage” phenomenon widely emphasized by the policy and science communities.^41^ As a region becomes more developed, the cost of pollution increases due to tightened local regulations. Businesses will seek alternative locations with laxer pollution restrictions to lower the cost of production, causing the shifting of pollution sources to the less developed regions, as revealed in this study.

### Lagging in Carbon Decoupling

The transfer of pollution to less developed regions can also be linked to the U-shaped relationship between pollution and economic development. Initially, pollution continues to increase with progress in economic development. Once a region is relatively developed, pollution will start to decline after a tipping point due to increased emphasis on environmental welfare. Such a relationship has been proven in global studies.^42^ The existence of a turning point in China’s CO_2_ emissions is also proven with Chinese historical data.^43^ The decoupling of carbon emissions and economic growth in the more developed coastal regions has also been verified by Zhou el al.^44^

**Figure 5:**
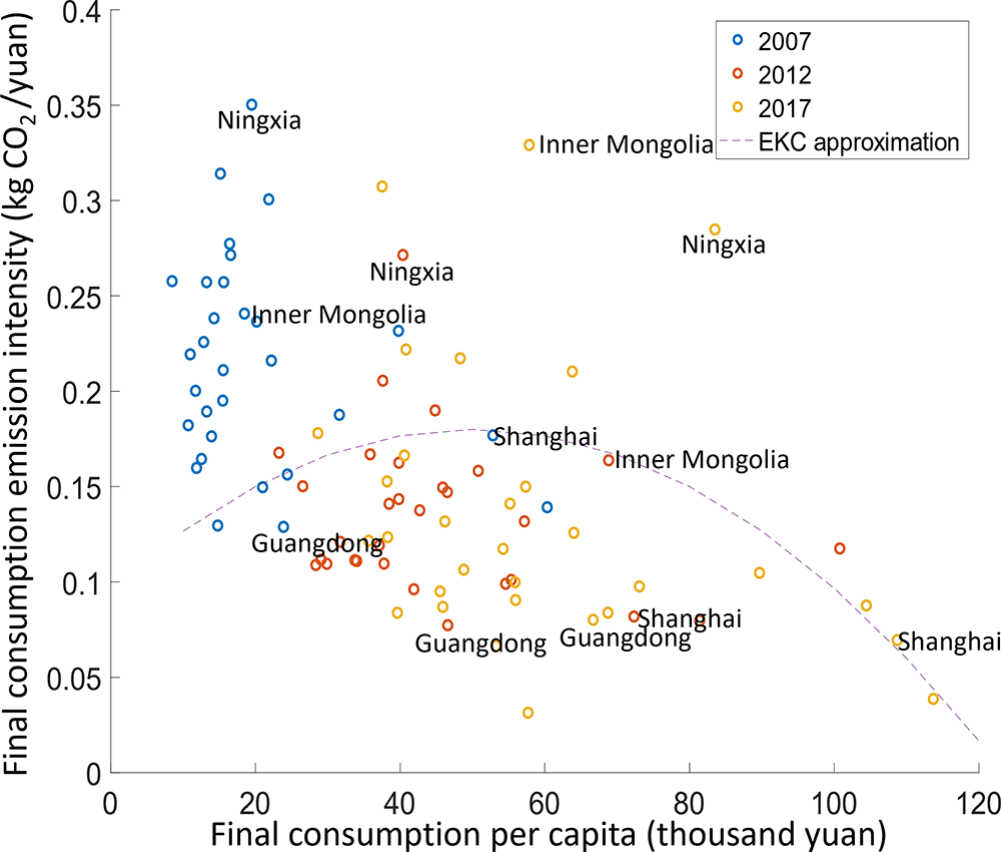
Final consumption emission intensities against final consumption per capita for China’s provinces. An approximated Environmental Kuznets Curve (EKC) is added as an illustration of the hypothetical relationship between environmental degradation and economic development.

For further analysis, CO_2_ intensities against consumption per capita are plotted in Figure 5. Doing so illustrates an inverted U-shaped relationship, coinciding with Environmental Kuznets Curve (EKC) theory. Although most EKC research adopts production-based accounting, some literatures also verify that consumption-based emissions may also follow the inverted U-shaped relationship with economic development.^45,46^ It is shown in Figure 5 that developed regions such as Beijing and Shanghai already exhibit a strong decoupling between final consumption and per unit emissions, but such a decoupling trend has yet to be discovered among less developed regions such as Inner Mongolia and Ningxia. Observation shows that the less developed regions of China still have great potential to improve their emission efficiencies.

An effective way to achieve CO_2_ emission mitigation is to target less developed and more emission-intensive regions, a strategy that has proven to be one of the most effective for CO_2_ emission mitigation.^7,47^ In fact, the Central Government of China has already realized the challenges in CO_2_ emission efficiencies faced by the less developed region of the northwest.^48^ In the upcoming 14th Five-Year Plan, policies targeted at the northwest regions of China have been devised to alleviate emission intensities to achieve China’s goal of peaking CO_2_ emissions by 2030.^49^ In addition, our study also quantitatively shows that the increasing of CO_2_ emissions embodied in the export from the northwest to other regions is a key contributor to the increasing polarization of CO_2_ emissions across China. Besides focused policies on the less developed regions only, the Central Government may also consider formalized mechanism to promote coordinated cross regional policies among both developing and developed regions. For instance, Clean Development Mechanism (CDM) has always been advocated in the international setting, but less attention has been diverted for CDM with countries’ borders. The Central Government may consider the implementation of similar mechanism to ensure more just allocation of emissions responsibilities among domestic players and less mitigation resource burden on the Central Government. In addition, financial tool may be an alternative for alleviating the inequality in CO_2_ emissions. Regulatory easing and subsidies for green bond issuance from less developed regions to the developed regions may also be a viable option.

### Trends with the World

However, CO_2_ emission mitigation is not only a domestic problem but also a global challenge that requires international coordination. Pollution transfer happens across borders between China and the world as well. Evidence supports our observation that the CO_2_ emissions embodied in China’s net exports to developed countries are already decreasing,^22^ shifting to the developing world.^50^ Such an observation serves as empirical evidence for our argument that a further shifting of the CO_2_ emissions embodied in exports from the less developed regions of China to the world in the near future is impending. Given the uncertainties imposed by China’s strict COVID-19 border control, further supply chain shifts from China to the rest of the world will be a very likely and imminent event.^51^

**Figure 6:**
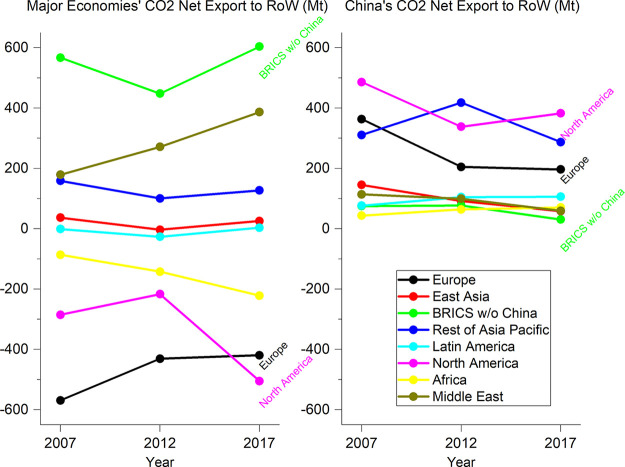
Net export of CO_2_ emissions from major world economic regions and China to the rest of the world other than China in 2007, 2012, and 2017. Please see Table S5 in Supporting Information for data result.

With the world MRIO table and emission inventory of the EXIOBASE database^34^ and the calculation by He and Hertwich,^52^Figure 6 is produced to show how CO_2_ emissions embodied in trade shifted from 2007 to 2017 across the world. To ensure the discrepancies between different data sources are minimized, we have normalized the global emissions calculated from EXIOBASE with the domestic CO_2_ emissions of China calculated in this study. In general, the CO_2_ emissions embodied in China’s net exports to the world decreased for all regions, which is in line with the findings of other studies. However, North America’s (a typical developed region) CO_2_ emissions embodied in its net imports show an increase from the world other than China from 2012 to 2017, while the net exports of embodied CO_2_ emissions from emerging economies also show an increase from 2012 to 2017.

Fortunately, the rapid development and deployment of clean technologies that were not available a decade ago may help developing countries achieve a clean and green reception of supply chains from China. In recent years, the cost of renewable energies has drastically decreased.^53^ Knowledge of the best practices for sustainable investment in energy infrastructures is becoming more available and is regarded as a larger priority by global policy makers.^54^ Recent evidence also shows that the exchange of clean energy technologies among countries may be able to put a stop to pollution outsourcing.^55^ In other words, having a variety of green technology choices provides us with an alternative future to the past of perpetual emission outsourcing. It could be the key for us to achieve an equally sustainable future for all countries, regardless of the relative levels of economic development and the overall levels of economic well-being.

## Data Availability

For the CO_2_ emission inventories, we adopted the CEADs database.^30^ The CEADs database (https://www.ceads.net/) is a widely utilized database for CO_2_ emissions in China. It follows IPCC Guidelines for National Greenhouse Gas Inventories when compiling its emission inventories.^31^ It gives a breakdown of CO_2_ emissions across Chinese provinces and sectors. We mapped the CEADs emission inventory in accordance with our MRIO table using a method applied by previous studies.^32,33^ Our MRIO table is compiled from the regional Input–Output tables published by the Bureau of Statistics of the respective provinces. Global flow of embodied CO_2_ emissions are calculated using data of EXIOBASE database.^34^

## Supporting Information Available

Data used for the production of figures and results analysis (PDF)
