# Supplementary material for: The Polarizing Trend of Regional CO2 Emissions in China and Its Implications
Source: Environ Sci Technol. 2023 Feb 28;57(11):4406–14. doi: 10.1021/acs.est.2c08052 (PMC10035028; doi:10.1021/acs.est.2c08052)
Supplement: Supplementary file 1 — es2c08052_si_001.pdf [file es2c08052_si_001.pdf]

Supplementary Information for:

The Polarising Trend of Regional CO_2 Emissions in China and Its Implications

Authors: Kehan He[1], Zhifu Mi[1*], Jin Zhang[2,3], Jinkai Li[2,4*], D'Maris Coffman[1]

[1] The Bartlett School of Sustainable Construction, University College London, London, WC1E 7HB, UK

[2] Center for Energy, Environment & Economy Research, Zhengzhou University, Zhengzhou 450001, China

[3] School of Public Policy and Management, Tsinghua University, Beijing 100084, China

[4] Center for Energy Economics and Sustainability, Peking University, Beijing 100871, China

[*] Corresponding Authors:

Zhifu Mi: z.mi@ucl.ac.uk

Jinkai Li: lijinkai@sina.com

Number of pages (Including this cover page): 8

Number of figures: 0

Number of tables: 5

This file includes:

Table S1. Province to Region Concordance.

Table S2. The Net Export of CO_2 Emissions Among the Eight Regions of China in 2007, 2012, and 2017.

Table S3. Consumption-Based CO_2 vs Final Consumption.

Table S4. Percentage of Consumption-Based CO_2 vs Percentage of Final Consumption.

Table S5. The Net Export of CO_2 Emissions Between China and Rest of the World (RoW) Regions in 2007, 2012, and 2017.

Table S1. Province to Region Concordance. It shows the specification of provinces assignment to regions

**Province**	**Region**	**Province**	**Region**
Beijing	Beijing–Tianjin	Hunan	Central
Tianjin	Beijing–Tianjin	Guangdong	South Coast
Hebei	North	Guangxi	Southwest
Shanxi	Central	Hainan	South Coast
Inner Mongolia	Northwest	Chongqing	Southwest
Liaoning	Northeast	Sichuan	Southwest
Jilin	Northeast	Guizhou	Southwest
Heilongjiang	Northeast	Yunnan	Southwest
Shanghai	Central Coast	Tibet	Southwest
Jiangsu	Central Coast	Shaanxi	Northwest
Zhejiang	Central Coast	Gansu	Northwest
Anhui	Central	Qinghai	Northwest
Fujian	South Coast	Ningxia	Northwest
Jiangxi	Central	Xinjiang	Northwest
Shandong	North	Taiwan	No data
Henan	Central	Hong Kong	No data
Hubei	Central	Macao	No data

Table S2. The Net Export of CO_2 Emissions Among the Eight Regions of China in 2007, 2012, and 2017.

Mt CO_2	2007	CNE	CBT	CNO	CCE	CCC	CSC	CNW	CSW	Total Domestic Export
Northeast	CNE	0.0	16.4	-5.1	5.8	19.1	8.1	-48.9	8.0	3.3
Beijing–Tianjin	CBT	-16.4	0.0	-40.3	-24.2	-1.0	0.3	-33.3	-2.8	-117.7
North	CNO	5.1	40.3	0.0	-19.3	77.1	21.2	-16.1	15.6	123.9
Central	CCE	-5.8	24.2	19.3	0.0	86.4	19.3	1.4	-8.1	136.8
Central Coast	CCC	-19.1	1.0	-77.1	-86.4	0.0	0.9	-40.9	-13.5	-235.0
South Coast	CSC	-8.1	-0.3	-21.2	-19.3	-0.9	0.0	-14.1	-35.0	-98.9
Northwest	CNW	48.9	33.3	16.1	-1.4	40.9	14.1	0.0	13.0	165.0
Southwest	CSW	-8.0	2.8	-15.6	8.1	13.5	35.0	-13.0	0.0	22.7
Mt CO_2	2012	CNE	CBT	CNO	CCE	CCC	CSC	CNW	CSW	Total Domestic Export
Northeast	CNE	0.0	13.9	-13.8	-4.5	0.3	7.0	-20.0	11.4	-5.7
Beijing–Tianjin	CBT	-13.9	0.0	-21.9	-27.0	-10.0	-0.6	-39.2	-4.5	-117.1
North	CNO	13.8	21.9	0.0	3.2	30.3	15.9	-38.1	13.0	59.9
Central	CCE	4.5	27.0	-3.2	0.0	38.6	26.6	-38.5	15.6	70.6
Central Coast	CCC	-0.3	10.0	-30.3	-38.6	0.0	14.5	-40.4	4.1	-81.1
South Coast	CSC	-7.0	0.6	-15.9	-26.6	-14.5	0.0	-25.6	-18.0	-107.1
Northwest	CNW	20.0	39.2	38.1	38.5	40.4	25.6	0.0	32.1	234.1
Southwest	CSW	-11.4	4.5	-13.0	-15.6	-4.1	18.0	-32.1	0.0	-53.7
Mt CO_2	2017	CNE	CBT	CNO	CCE	CCC	CSC	CNW	CSW	Total Domestic Export
Northeast	CNE	0.0	7.0	3.9	1.5	13.9	11.6	-24.6	4.7	18.0
Beijing–Tianjin	CBT	-7.0	0.0	-1.6	-6.8	-2.3	-0.1	-18.9	-2.9	-39.7
North	CNO	-3.9	1.6	0.0	-4.4	1.9	1.8	-22.6	-0.8	-26.4
Central	CCE	-1.5	6.8	4.4	0.0	15.0	15.8	-36.0	5.3	9.8
Central Coast	CCC	-13.9	2.3	-1.9	-15.0	0.0	3.2	-49.5	-6.3	-81.1
South Coast	CSC	-11.6	0.1	-1.8	-15.8	-3.2	0.0	-48.3	-8.0	-88.5
Northwest	CNW	24.6	18.9	22.6	36.0	49.5	48.3	0.0	30.3	230.2
Southwest	CSW	-4.7	2.9	0.8	-5.3	6.3	8.0	-30.3	0.0	-22.2

Table S3. Consumption-Based CO_2 vs Final Consumption. The consumption-based CO_2 emissions, final consumptions, CO_2 emission intensities, and CO_2 net export of 31 provinces in 2007, 2012, and 2017. The deeper red in CO_2 net export means the larger negative net export, while the deeper blue in CO_2 net export means the larger positive net export.

	2007				2012				2017			
	CBA CO_2 emission intensity (kg/yuan)	Final Consumption (trillion Yuan)	CBA CO_2 (Mt)	CO_2 net export (Mt)	CBA CO_2 emission intensity (kg/yuan)	Final Consumption (trillion Yuan)	CBA CO_2 (Mt)	CO_2 net export (Mt)	CBA CO_2 emission intensity (kg/yuan)	Final Consumption (trillion Yuan)	CBA CO_2 (Mt)	CO_2 net export (Mt)
Beijing	0.14	1011.1	140.7	-75.5	0.08	1684.5	134.3	-69.0	0.04	2468.5	95.4	-27.0
Tianjin	0.23	443.5	102.7	-42.2	0.12	1424.0	167.4	-48.1	0.09	1626.1	142.5	-12.7
Hebei	0.24	991.4	236.2	128.2	0.17	2612.9	436.1	119.5	0.22	3068.9	681.1	4.9
Shanxi	0.31	514.3	161.5	92.1	0.21	1357.8	279.1	101.8	0.31	1388.7	426.8	44.3
Inner Mongolia	0.24	448.7	108.0	159.2	0.16	1713.5	280.6	191.1	0.33	1462.9	481.5	145.6
Liaoning	0.24	866.6	205.0	55.0	0.13	2509.4	330.7	37.9	0.22	2110.6	458.6	2.9
Jilin	0.30	596.3	179.3	-45.7	0.16	1397.2	221.1	-17.7	0.14	1499.2	211.5	-13.6
Heilongjiang	0.26	597.0	153.5	-6.0	0.16	1527.0	248.1	-25.9	0.13	1749.9	230.5	28.6
Shanghai	0.18	1090.1	192.8	-105.7	0.08	1721.4	140.9	-9.4	0.07	2628.6	182.7	-5.3
Jiangsu	0.16	1885.7	294.8	-11.5	0.10	4383.6	443.1	-6.7	0.10	7199.6	754.1	-36.7
Zhejiang	0.19	1629.0	305.6	-117.9	0.10	2989.2	296.0	-65.0	0.10	4133.3	403.6	-39.2
Anhui	0.20	717.1	143.6	1.1	0.11	1740.7	194.5	47.5	0.15	2389.4	364.9	-9.3
Fujian	0.15	758.4	113.5	-20.0	0.10	1570.3	151.0	1.0	0.08	2687.6	225.4	-0.9
Jiangxi	0.26	578.6	148.8	-55.4	0.11	1276.8	139.0	-19.5	0.12	1769.3	218.4	-2.8
Shandong	0.22	2079.4	449.3	-4.3	0.15	4506.0	662.8	-59.6	0.13	6405.2	805.6	-31.3

Henan	0.18	1303.7	229.9	79.7	0.12	3491.4	416.4	-4.4	0.11	4664.9	496.4	-23.1
Hubei	0.20	882.6	172.2	14.7	0.14	2223.2	313.4	-24.4	0.09	3303.1	298.9	6.5
Hunan	0.19	843.4	159.7	4.6	0.11	2256.9	250.5	-30.3	0.10	3122.7	296.8	-5.8
Guangdong	0.13	2306.4	297.2	-78.5	0.08	4934.6	381.5	-99.8	0.08	7447.3	596.8	-95.6
Guangxi	0.16	565.6	90.4	-3.6	0.11	1579.4	176.0	-22.5	0.12	1741.9	211.9	2.9
Hainan	0.13	124.9	16.2	-0.4	0.11	334.9	36.7	-8.3	0.07	493.0	32.8	7.9
Chongqing	0.21	437.6	92.4	-16.2	0.14	1172.5	168.2	-36.6	0.10	1718.5	171.6	-23.2
Sichuan	0.16	1019.5	167.7	-12.6	0.11	2412.5	264.2	-24.2	0.08	3289.0	275.7	10.3
Guizhou	0.26	308.0	79.4	42.6	0.17	810.0	135.9	42.7	0.17	1452.8	241.6	-3.5
Yunan	0.18	483.5	88.1	12.5	0.12	1477.1	178.8	-13.2	0.09	2203.7	191.5	-7.5
Tibet	-	-	-		-	-	-		0.03	194.4	6.1	-1.3
Shaanxi	0.23	476.4	107.6	-1.9	0.14	1602.9	220.5	-30.1	0.12	2079.6	244.1	6.3
Gansu	0.22	279.9	61.4	7.9	0.15	684.1	102.7	20.1	0.18	752.0	133.9	7.5
Qinghai	0.28	90.7	25.2	-6.7	0.15	263.0	39.3	-4.4	0.15	342.9	51.4	-1.7
Ningxia	0.35	118.9	41.6	10.0	0.27	261.4	71.0	44.4	0.28	569.5	162.2	11.0
Xinjiang	0.27	347.3	94.3	-3.4	0.19	1001.1	190.2	13.0	0.21	1559.7	328.0	61.4

Table S4. Percentage of Consumption-Based CO_2 vs Percentage of Final Consumption. The percentage share of consumption-based CO_2 emissions and final consumptions of 31 provinces in 2007, 2012, and 2017. Differences in percentage points between consumption-based CO_2 emissions and final consumptions are also presented. The deeper red in differences in percentage points means the larger share of consumption-based CO_2 emissions than final consumption, while the deeper blue in differences in percentage points means the larger share of final consumption than consumption-based CO_2 emissions.

	% of Final Consumption			% of CBA CO_2 emissions			Differences in %			Changes in %
	2007	2012	2017	2007	2012	2017	2007	2012	2017	2007 - 2017
Beijing	4.2%	3.0%	3.2%	3.0%	1.9%	1.0%	1.2%	1.1%	2.2%	0.9%
Tianjin	1.9%	2.5%	2.1%	2.2%	2.4%	1.5%	-0.3%	0.1%	0.6%	0.9%
Hebei	4.2%	4.6%	4.0%	5.1%	6.2%	7.2%	-0.9%	-1.6%	-3.3%	-2.4%
Shanxi	2.2%	2.4%	1.8%	3.5%	3.9%	4.5%	-1.3%	-1.6%	-2.7%	-1.4%
Inner Mongolia	1.9%	3.0%	1.9%	2.3%	4.0%	5.1%	-0.4%	-1.0%	-3.2%	-2.8%
Liaoning	3.6%	4.4%	2.7%	4.4%	4.7%	4.9%	-0.8%	-0.3%	-2.1%	-1.4%
Jilin	2.5%	2.5%	1.9%	3.8%	3.1%	2.2%	-1.3%	-0.7%	-0.3%	1.0%
Heilongjiang	2.5%	2.7%	2.3%	3.3%	3.5%	2.4%	-0.8%	-0.8%	-0.2%	0.6%
Shanghai	4.6%	3.0%	3.4%	4.1%	2.0%	1.9%	0.4%	1.0%	1.5%	1.0%
Jiangsu	7.9%	7.7%	9.3%	6.3%	6.3%	8.0%	1.6%	1.4%	1.3%	-0.3%
Zhejiang	6.8%	5.3%	5.3%	6.6%	4.2%	4.3%	0.3%	1.1%	1.0%	0.8%
Anhui	3.0%	3.1%	3.1%	3.1%	2.8%	3.9%	-0.1%	0.3%	-0.8%	-0.7%
Fujian	3.2%	2.8%	3.5%	2.4%	2.1%	2.4%	0.8%	0.6%	1.1%	0.3%
Jiangxi	2.4%	2.2%	2.3%	3.2%	2.0%	2.3%	-0.8%	0.3%	0.0%	0.7%
Shandong	8.7%	7.9%	8.3%	9.6%	9.4%	8.5%	-0.9%	-1.5%	-0.3%	0.6%
Henan	5.5%	6.1%	6.0%	4.9%	5.9%	5.3%	0.5%	0.2%	0.8%	0.2%
Hubei	3.7%	3.9%	4.3%	3.7%	4.4%	3.2%	0.0%	-0.5%	1.1%	1.1%
Hunan	3.5%	4.0%	4.0%	3.4%	3.5%	3.1%	0.1%	0.4%	0.9%	0.8%
Guangdong	9.7%	8.7%	9.6%	6.4%	5.4%	6.3%	3.3%	3.3%	3.3%	0.0%
Guangxi	2.4%	2.8%	2.2%	1.9%	2.5%	2.2%	0.4%	0.3%	0.0%	-0.4%
Hainan	0.5%	0.6%	0.6%	0.3%	0.5%	0.3%	0.2%	0.1%	0.3%	0.1%
Chongqing	1.8%	2.1%	2.2%	2.0%	2.4%	1.8%	-0.1%	-0.3%	0.4%	0.5%
Sichuan	4.3%	4.2%	4.2%	3.6%	3.7%	2.9%	0.7%	0.5%	1.3%	0.6%
Guizhou	1.3%	1.4%	1.9%	1.7%	1.9%	2.6%	-0.4%	-0.5%	-0.7%	-0.3%
Yunan	2.0%	2.6%	2.8%	1.9%	2.5%	2.0%	0.1%	0.1%	0.8%	0.7%
Tibet			0.3%			0.1%	0.0%	0.0%	0.2%	0.2%
Shaanxi	2.0%	2.8%	2.7%	2.3%	3.1%	2.6%	-0.3%	-0.3%	0.1%	0.4%
Gansu	1.2%	1.2%	1.0%	1.3%	1.5%	1.4%	-0.1%	-0.3%	-0.5%	-0.3%
Qinghai	0.4%	0.5%	0.4%	0.5%	0.6%	0.5%	-0.2%	-0.1%	-0.1%	0.1%
Ningxia	0.5%	0.5%	0.7%	0.9%	1.0%	1.7%	-0.4%	-0.5%	-1.0%	-0.6%
Xinjiang	1.5%	1.8%	2.0%	2.0%	2.7%	3.5%	-0.6%	-0.9%	-1.5%	-0.9%

Table S5. The Net Export of CO_2 Emissions Between China and Rest of the World (RoW) Regions in 2007, 2012, and 2017.

	2007		2012		2017	
CO_2 Net Export (Mt)	to RoW	to China	to RoW	to China	to RoW	to China
Europe	-569.2	-363.6	-431.0	-204.8	-419.3	-196.6
North America	-285.1	-486.2	-216.7	-338.3	-505.0	-382.6
East Asia	36.9	-145.4	-3.4	-91.6	25.7	-58.3
China	1615.2	0.0	1398.1	0.0	1191.5	0.0
BRICS w/o China	566.9	-75.2	448.1	-77.3	603.7	-30.7
Rest of Asia Pacific	158.7	-310.7	100.6	-417.9	127.0	-287.3
Latin America	-0.9	-76.7	-26.7	-104.7	3.2	-106.1
Africa	-86.2	-43.5	-142.3	-63.9	-221.9	-70.6
Middle East	178.9	-114.1	271.4	-99.6	386.6	-59.2
